# Ansa pancreatica: a rare cause of acute recurrent pancreatitis

**DOI:** 10.11604/pamj.2020.37.202.23218

**Published:** 2020-10-29

**Authors:** Abdelilah El Bakouri, Othmane El Yamine, Mounir Bouali, Fatima Zahra Bensardi, Khalid El hattabi, Abdelaziz Fadil

**Affiliations:** 1 Service des Urgences Chirurgicales Viscérales, CHU Ibn Rochd, Université Hassane II, Faculté de Médecine et de Pharmacie (FMPC), Casablanca, Maroc

**Keywords:** Ansa pancreatica, acute pancreatitis, magnetic resonance cholangiopancreatography, sphincterotomy

## Abstract

Acute pancreatitis is an inflammation of the pancreas that can be caused in rare situations by ansa pancreatica, it is a rare anatomic variation of the pancreatic ducts. It is a communication between the main pancreatic duct (Wirsung) and the accessory pancreatic duct (Santorini). We report a case of the patient, in a 44-year-old, non-alcoholic, hospitalized for acute pancreatitis stage C of Baltazar. A magnetic resonance Cholangiopancreatography (MRCP) was performed which showed a gallstone and ansa pancreatica, than an endoscopic retrograde cholangiopancreatography (ERCP) revealed an ansa pancreatica with a common bile duct clear, a sphincterotomy of the major papilla was performed. It is still not clear whether the presence of these two pathologies is a coincidence or if the ansa pancreatica is the cause of acute pancreatitis. New studies are necessary to clarify these points.

## Introduction

Acute pancreatitis (AP), an inflammatory disease of the pancreas, is a common cause of gastrointestinal hospitalizations [1]. Gallstones, alcohol intake are the most common causes of AP. Variations in pancreatic duct (PD) anatomy have also been shown to play a role in some cases of AP. Typically, the downstream pancreatic duct system within the head is made up of the ducts of Wirsung and Santorini (the accessory pancreatic duct). There are many different types and anatomic variations of the pancreatic ducts, ansa pancreatica is a rare anatomic variation, with a reported prevalence of 1.1% [2,3]. Recently, the ansa pancreatica has been considered as a predisposing factor in patients with idiopathic acute pancreatitis [4]. We report the observation of a patient with acute pancreatitis in connection with an Ansa pancreatica.

## Patient and observation

**Figure 1:**
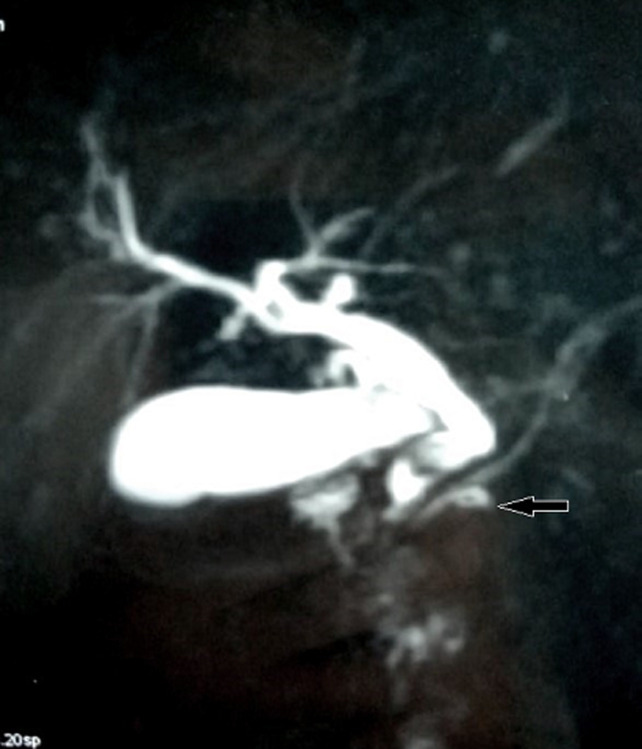
MRI showing a dilatation of the princpale biliary tract upstream of a lithiasis with the appearance of a loop at the level of the wirsung canal evoking an ansa pancreatica

**Figure 2:**
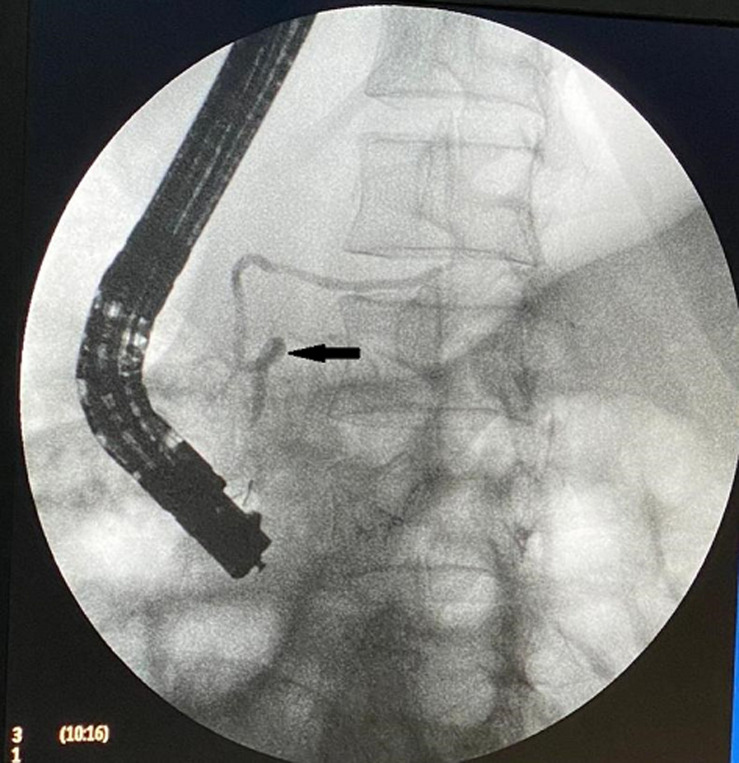
ERCP showing aspect in favor of an ansa pancreatica

A 44-year-old female with a past medical history of an episode of AP one year before of unknown etiology, non alcoholic. His pain was located in epigastrium radiating to the back for three days, associated with vomiting. On admission, the patient was hemodynamically and respiratory stable, with epigastric sensitivity, the serum lipase level was 560U/L (normal range, 13-60 U/L), CRP rate was high at 130 mg/l, the abdominal CT scan showed pancreatitis stage C of Baltazar with a common bile duct dilated to 13 mm, liver function tests was disturbed. The patient was also found to have normal calcium, triglyceride, and IgG4 level. He was not taking any medications. Since it is recurrent pancreatitis, we did magnetic resonance cholangiopancreatography (MRCP) which showed a gallbladder stones and dilation of the common bile duct to 10 mm with a sudden stop of low choledochal duct interpreted as lithiasis, with an aspect of a loop at the level of the accessory pancreatic duct evoking an ansa pancreatica (Figure 1); than an endoscopic retrograde cholangiopancreatography (ERCP) revealed an ansa pancreatica with a common bile duct clear without lithiasis (Figure 2), a sphincterotomy of the major papilla was performed to lower the downstream tension. Cholecystectomy was performed, the post-operative was simple, the anatomopathological examination of the part did not show any neoplastic lesion.

## Discussion

The pancreatica ansa is a rare type of pancreas ductal variation first described by Dawson in 1961. It is an accessory pathway between the Wirsung Canal and an accessory pancreatic duct that does not have a normal junction with the former. It would be formed by the junction of the lower branches of the main and accessory pancreatic ducts. In this configuration, the minor papilla appears to be most often permeable [5]. Dawson and Langman called ansa pancreatica the formation in which communication between the lower branches of the Wirsung and Santorini ducts overrides the absence of normal junction of these ducts; this induces a looped image [6], and Simkins hypothesized that these aberrant junctions are the result of initial plexus development of the pancreatic ducts during embryogenesis, with only those in which sufficient flow ultimately persists; however, there are no embryological studies to support this hypothesis [7]. Kamisawa uses this “flow theory” to describe short or long Santorini canals whose formation would depend on the anterograde or retrograde flow in the Santorini canal [8]. There is poor drainage of pancreatic juice in ansa pancreatica since the main pancreatic duct and side branch meets at a sloping angle, making patients vulnerable to pancreatitis [9]

**Figure 3:**
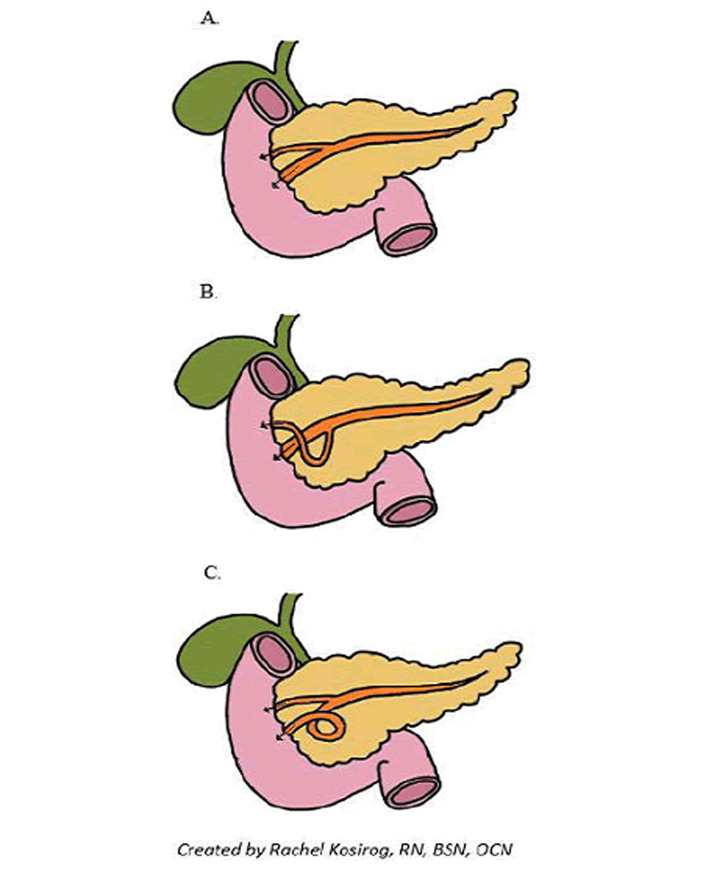
normal pancreatic duct anatomy (A) and different types of ansa pancreatica (B,C)

Ansa pancreatica has been described in two forms. The first is where the duct of Santorini forms an S-shape en route to the duct of Wirsung, in the second form, a looping branch is seen within the duct of Wirsung as it joins the duct of Santorini [10] (Figure 3). There are not many studies that treat the association of acute pancreatitis and ansa pancreatica, and its prevalence is not well established. Only three articles commented on the prevalence of ansa pancreatica with acute pancreatitis. one Japanese study that consisted of a community-based cohort of 587 patients who attended a paid medical examination in which, among other things, an MRCP was performed on all subjects, they found that 0.85% of the patients suffered from pancreatitis ansa [11]. This study could not be applied to the general population in other parts of the world since it included only Japanese subjects. In this study, a statistically significant correlation between ansa pancreatica and the onset of acute recurrent pancreatitis was established. Also, MRCP may underestimate the actual incidence of ansa pancreatica Ishii *et al*. looked at all of the ERCPs done at their institution in Tokyo over 24-years, and they found that out of the 3040 patients that had ERCPs only 15 of these had ansa pancreatica with a rate of 0.5% [12]. In a Turkish population based, retrospective study of 1158 MRCPs of people with a suspected biliary or pancreatic disease, the ansa pancreatica incidence was found to be 1.2% [13], in the results of this study ansa pancreatica might be considered a relevant factor to the onset of chronic pancreatitis. According to Hiroshi, about 7% of patients with an ansa pancreatica have acute pancreatitis [12].

Magnetic resonance cholangiopancreatography (MRCP) and endoscopic retrograde cholangiopancreatography (ERCP) are revolutionizing the study by becoming the preferred diagnostic tests to identify the anatomical variant when it becomes symptomatic [9] and are essential for the etiological assessment of recurrent acute non-biliary pancreatitis [14]. No statistically significant association was detected between pancreatitis ansa and the demographic characteristics of the subjects examined in any of the studies found, either based on examination of cadavers, ERCP-MRCP assessment or a combination of methods [15]. Treatment strategies are not well described in the literature, but it is reasonable to say that because of the findings described by Kamisawa and Dawson, relief of downstream pressure by sphincterotomy may be beneficial [6,8,10] In our case the patient has an ansa pancreatica associated with a lithiasis pathology, we performed a sphincterotomy and then a cholecystectomy in order to avoid a recurrence of another episode of pancreatitis either by biliary lithiasis or by an ansa pancreatica.

## Conclusion

Ansa pancreatica may be considered a predisposing factor for acute idiopathic pancreatitis. Magnetic resonance cholangiopancreatography (MRCP) allows for diagnosis, sphincterotomy to relieve downstream tension, and an option to treat and prevent recurrence of pancreatitis caused by ansa pancreatica. Further studies of a randomized nature are needed to properly study the risk of pancreatic-biliary disease in patients with this anatomical malformation of the pancreatic duct.
